# Expression and function of angiopoietin-1 in breast cancer

**DOI:** 10.1054/bjoc.2000.1437

**Published:** 2000-11

**Authors:** A J Hayes, W-Q Huang, J Yu, P C Maisonpierre, A Liu, F G Kern, M E Lippman, S W McLeskey, L-Y Li

**Affiliations:** Department of Oncology, Department of Biostatistics, Georgetown University Medical Center, 3970 Reservoir Road, NW RB/E301, Washington, DC, 20007; Regeneron Pharmaceuticals Inc, Tarrytown, NY, 10591; Drug Discovery Division, Southern Research Institute, Birmingham, AL, 35255, USA

**Keywords:** angiogenesis, angiopoietin, neovascularization, breast cancer, gene expression, tumorigenesis

## Abstract

Angiopoietin-1 (Ang1) has been shown to act as an angiogenic promoter in embryonic angiogenesis by promoting vascular branching, pericyte recruitment and endothelial survival. We have investigated the role of Ang1 in tumour neovascularization under clinical conditions and in animal models. The expression of Ang1 in clinical breast cancer specimens was analysed by using laser-capture microdissection and reverse transcriptase-linked polymerase chain reaction (RT-PCR) on RNA isolated from the samples. Despite the expression of Ang1 in many human breast cancer cell lines, the gene was expressed in only three of 21 breast cancer clinical specimens, even though its receptor, Tie2, is abundant in the vasculature of all of these tumours. Ang1 was then overexpressed in a human breast cancer cell line (MCF-7) on its own and in conjunction with FGF1, an angiogenic factor shown to be able to increase the tumorigenicity of MCF-7 cells. High concentrations of Ang1 were produced in the conditioned media of the transfected cells (range 156–820 ng ml^–1^). However, in contrast to its physiological role as promoter of angiogenesis, overexpression of Ang1 did not enhance tumour growth, but instead caused up to a 3-fold retardation of tumour growth (*P* = 0.003). © 2000 Cancer Research Campaign


*British Journal of Cancer* (2000) **83**(9), 1154–1160
© 2000 Cancer Research Campaign
doi: 10.1054/ bjoc.2000.1437, available online at http://www.idealibrary.com on IDEAL®

### Expression and function of angiopoietin-1 in breast cancer

**AJ Hayes[1], W-Q Huang[1], J Yu[1], PC Maisonpierre[3], A Liu[2], FG Kern[4], ME Lippman[1], SW McLeskey[1] and L-Y Li[1]**

[1]Department of Oncology; [2]Department of Biostatistics, Georgetown University Medical Center, 3970 Reservoir Road, NW, RB/E301, Washington DC 20007; [3]Regeneron Pharmaceuticals Inc, Tarrytown NY 10591; [4]Drug Discovery Division, Southern Research Institute, Birmingham AL 35255, USA

**Summary** Angiopoietin-1 (Ang1) has been shown to act as an angiogenic promoter in embryonic angiogenesis by promoting vascular branching, pericyte recruitment and endothelial survival. We have investigated the role of Ang1 in tumour neovascularization under clinical conditions and in animal models. The expression of Ang1 in clinical breast cancer specimens was analysed by using laser-capture microdissection and reverse transcriptase-linked polymerase chain reaction (RT-PCR) on RNA isolated from the samples. Despite the expression of Ang1 in many human breast cancer cell lines, the gene was expressed in only three of 21 breast cancer clinical specimens, even though its receptor, Tie2, is abundant in the vasculature of all of these tumours. Ang1 was then overexpressed in a human breast cancer cell line (MCF-7) on its own and in conjunction with FGF1, an angiogenic factor shown to be able to increase the tumorigenicity of MCF-7 cells. High concentrations of Ang1 were produced in the conditioned media of the transfected cells (range 156–820 ng $ml^{-1}$). However, in contrast to its physiological role as promoter of angiogenesis, overexpression of Ang1 did not enhance tumour growth, but instead caused up to a 3-fold retardation of tumour growth ($P$ = 0.003). © 2000 Cancer Research Campaign

**Keywords:** angiogenesis; angiopoietin; neovascularization; breast cancer; gene expression; tumorigenesis

Tumours undergo a critical transition from an avascular to a vascular stage by invoking an angiogenic response from host vasculature (Folkman, 1971). To do this the cancer cells elaborate growth factors and cytokines that act upon the surrounding endothelial cells (Hanahan and Folkman, 1996) which induce vascular sprouting and new blood vessel formation. Certain of these factors, such as fibroblast growth factor-1 (FGF1), may have both paracrine and autocrine effects on tumour growth because of the distribution of their receptors on tumour cells and other stromal cells, as well as endothelial cells (Lehtola et al, 1992). Other factors such as vascular endothelial cell growth factor (VEGF), target only endothelial cells because the expression of their receptors is restricted to endothelial cells (de Vries et al, 1992; Terman et al, 1992).

Angiopoietin-1 (Ang1) is a recently described growth factor whose target cells are also endothelial cells, because the expression of the receptor, Tie2 (Tek), is restricted essentially to endothelial cells (Dumont et al, 1993; Maisonpierre et al, 1993). Transgenic animal studies indicate that Ang1 is central to embryonic vascular development (Sato et al, 1995; Suri et al, 1996; 1998; Maisonpierre et al, 1997; Thurston et al, 1999). Ang1 knock-out animals die in utero because of specific vascular deficits. These include failure of the primitive capillary plexus to branch appropriately and failure of the endothelial cells to form stable associations with surrounding basement membranes and pericytes. Knock-out of the Tie2 gene gave rise to essentially the same phenotype. Overexpression of Ang1 in the skin of transgenic animals causes an increase in the number, size and branching complexity of dermal vessels. In vitro, Ang1 protects against endothelial cell apoptosis, stabilizes endothelial tubules and induces vascular sprouting and tubule formation in collagen (Koblizek et al, 1998; Hayes et al, 1999; Holash et al, 1999; Papapetropoulos et al, 1999; Thurston et al, 1999). In vivo Ang1 synergizes with VEGF to promote corneal neovascularization (Asahara et al, 1998). Together these findings all suggest that Ang1 acts physiologically as an angiogenic promoter in the developing vascular system, in a coordinated process involving other angiogenic factors such as VEGF (Shalaby et al, 1995; Carmeliet et al, 1996; Ferrara et al, 1996).

We investigated whether Ang1 would serve an analogous function in tumours by promoting vascularization. Ang1 expression levels were assessed in breast cancer cell lines and in breast cancer specimens. The gene was then overexpressed in a breast cancer cell line (MCF-7) by itself or together with FGF1. Our data indicate that Ang1 is rarely expressed by breast cancer cells in clinical samples. Moreover, Ang1 not only fails to enhance xenograft tumour growth in the MCF-7 tumour model, but appears to act in inhibitory capacity in this model. Possible reasons for the apparently contradictory roles for Ang1 in physiological and tumour angiogenesis are discussed.

### METHODS

### Cell culture

The MCPX cell line is a sub-line of ML-20 cells which were derived from MCF-7 cells by transfection with β-galactosidase (lacZ) (Kurebayashi et al, 1993; McLeskey et al, 1993; 1998). The α18 cells, referred to as Clone 18 previously, were derived from ML-20 cells by transfection with FGF1 (Zhang et al, 1997). Cell lines were cultured in 5% FBS, IMEM, 4 mM glutamine (Biofluids, Rockville MA, USA).

*Received 17 March 2000*
*Revised 3 July 2000*
*Accepted 11 July 2000*

*Correspondence to:* L-Y Li

### Immuno-histochemistry for Tie2 and vWF

Frozen sections (5 μm) were immuno-stained with monoclonal antibodies to Tie2 (a gift from Dr Kevin Peters, Procter and Gamble, Cincinnati OH, USA), and to von Willebrand factor (Boehringer Mannheim, Indianapolis IN, USA). Sections were fixed in acetone at –20°C for 10 min, incubated in 0.3% hydrogen peroxide in methanol, blocked in 5% BSA (Sigma, Milwaukee WI, USA) 2.5% normal horse serum (Vector Labs, Burlingame CA, USA) in PBS then incubated with antibody in blocking solution. Sections were washed in PBS, then incubated for 1 h with a 1:5000 dilution of a biotinylated polyclonal antibody against mouse IgG and developed using a Vector ABC kit (Vector Labs), then counter-staining with haematoxylin.

### Laser-capture microdissection

Frozen tumour samples and adjacent normal breast tissue were used for laser-capture microdissection using previously described methods (Emmert-Buck et al, 1996), utilizing a Pixcell laser-capture microdissection system (Arcturus Engineering, Mountain View CA, USA). Clinical sections, immuno-stained for Tie2 and vWF, were examined to identify areas of high microvessel density. Microvessel counting was performed using standard techniques (Weidner, 1995). Unmounted serial sections stained with eosin, or with haematoxylin and eosin, were then examined to locate parallel areas. A Capsure Cap (Arcturus Engineering) was then placed onto the section over a 30 μm diameter capture area comprising either tumour cells in areas of high microvessel density, or epithelial cells in normal breast acini. Upon laser capture, cells in 50 areas were transferred into a 0.5 ml Eppendorf vial, yielding 500–1000 cells per sample. Total RNA was retrieved using a Micro RNA Isolation kit (Stratagene, La Jolla CA, USA) and RNA corresponding to that collected from approximately 250 cells was used as a template for subsequent RT-PCR.

### Reverse transcriptase-linked polymerase chain reaction (RT-PCR)

The primer sequences for Ang1 are: 5′-CAACTGGAGC TGATG-GACAC A-3′ (sense) and 5′-ACTGCCTCTG ACTGGTAATG G-3′ (antisense) and span base pairs 1060–1420 of Ang1 mRNA. The primer sequences for VEGF are 5′-GCCTTGCCTT GCTGCTCTAC-3′ (sense) and 5′-AATGCTTTCT CCGCT-CTGA-3′ (antisense) spanning base pairs 48–473. The primer sequences for glyceraldehyde-3-phosphate dehydrogenase (GAPDH) are 5′-AAGGTGAAGG TCGGAGTCAA CG-3′ (sense) and 5′-TGGTGGTGCA GGAGGCATTG C-3′ (antisense) spanning base pairs 40–496. The GAPDH primers span introns 1–5 in the genomic sequence. Total RNA collected from a panel of breast cancer cell lines was supplied by Dr Anke Schultke, Lombardi Cancer Center and 0.1 μg was used as a template. RT-PCR analysis was performed using reagents supplied in a GeneAmp RNA PCR core kit (Perkin Elmer, Wellesley MA, USA), with a Perkin Elmer GeneAmp 2400 PCR system. The DNA was transferred onto a Nytran nylon membrane (Schleicher & Schuell, Keene NH, USA). Southern hybridization was performed using oligonucleotide nested primers end-labelled with $\gamma$-$^{32}$P-dATP (Amersham, Piscataway NJ, USA). The sequences of the nested primers were: for Ang1, 5′-AGACTGTGCA TGTATATC-3′; for VEGF, 5′-CAATGACGAG GGCCTGGAGT-3′; and for GAPDH, 5′-GTCTTCACCA CCATGGAGAA-3′.

### Transfection of the cancer cells with Ang1

The plasmid, jFE14/Ang1 is derived from the pSRα plasmid which contains an HTLV-1 promoter (Takebe et al, 1988). Full-length human Ang1 cDNA was cloned into this plasmid at two BstX 1 sites. Co-transfection was performed with the pcDNA3.1 Zeo plasmid (Invitrogen) which confers resistance to the antibiotic Zeocin (250 mg ml$^{-1}$), using Lipofectamine Plus reagent (GibcoBRL, Rockville MD, USA).

### Western slot blot analysis

Conditioned media were mixed (1:1) with DPBS containing 0.01% CHAPS (Sigma) and loaded in duplicate onto a Bio-dot SF slot blot apparatus (Bio-Rad, Hercules CA, USA), with Hybond ECL nitrocellulose membrane. After blocking, the membrane was incubated a rabbit polyclonal antibody against Ang1, developed at Regeneron Pharmaceuticals (Tarrytown NY, USA), in TBS, 0.1% Tween-20, 1.25% BSA, washed, then incubated with a horseradish peroxidase-conjugated anti-rabbit IgG antibody and developed using ECL chemiluminescence (Amersham)

### Tie2 phosphorylation assay

Tie2-transfected fibroblasts were treated for 5 min with conditioned media then immunoprecipitated for phosphorylated Tie-2 as described previously (Maisonpierre et al, 1997). Cells were lysed in RIPA buffer containing 1% Nonidet P40, 0.5% sodium deoxycholate, 0.1% SDS plus protease- and phosphatase-inhibitors. Tie2 was immunoprecipitated using an anti-myc monoclonal antibody, 9E10 (Sigma) and protein G-Sepharose beads. Samples were analysed by standard Western blotting protocols, including resolution by reducing SDS-PAGE and electrotransfer to PVDF membranes. The amount of total Tie2 receptor and autophosphorylated Tie2 receptor was detected, respectively, by incubating replicate membranes with 9E10 or with the anti-phosphotyrosine-specific monoclonal antibody, 4G10 (Upstate Biotechnology, Lake Placid NY, USA) visualized with a HRP-conjugated secondary antibody.

### BIAcore analysis

Conditioned media were analysed for Ang1 with a BIAcore biosenser (Pharmacia, Piscataway NJ, USA) utilizing a CM5 BIAcore chip surface that had been covalently coupled with recombinant soluble Tie2-Fc as described previously (Davis et al, 1996). To control for the specificity of ligand binding, media were pre-incubated with 20 μg ml$^{-1}$ of excess soluble Tie2-Fc or two irrelevant soluble receptors. Only soluble Tie2-Fc competed for Ang1 binding to the chip surface.

### Northern blotting analysis for FGF1 expression

An FGF1 cDNA probe was labelled with $\alpha^{32}$P-dCTP (Amersham) using a Random-Primers DNA labelling system (GibcoBRL) and used in Northern analyses of total RNA as described previously (Zhang et al, 1997).

*© 2000 Cancer Research Campaign*

### Tumorigenicity assays

NCR (nu/nu) athymic female 4–6-week-old mice (Taconic Farms Inc, Germantown NY, USA) were supplemented with subcutaneously embedded 0.72 mg oestrogen pellets then inoculated subcutaneously in the mammary fat pads with $5 \times 10^6$ cells in 0.2 ml IMEM. Tumour volumes (length × width × height) were measured two times a week in a blinded manner.

### Statistical analyses

Generalized linear mixed effects models were used to estimate tumour growth rates. The analysis was performed using SAS PROC MIXED procedure according to SAS/STAT User's Guide (SAS Institute Inc, Cary NC, USA). Plots of tumour sizes vs time for the MPCX tumour growth data revealed an exponential growth pattern. Plots of tumour size vs time for the α18 tumour growth data indicated the growth rate in some cell lines was negative to some time-points and then became positive, suggesting that an overall growth rate was not appropriate to summarize the growth pattern. For these data, we described the growth pattern for each cell line at certain time-points and compared the growth rates at these time-points using repeated analysis of variance.

### RESULTS

### Ang1 expression in breast cancer cell lines

We analysed Ang1 expression in a panel of 19 breast cancer cell lines by RT-PCR analysis (Figure 1). Ang1 transfected CHO cells were used as a positive control. PCR products were subjected to agarose gel electrophoresis and visualized with ethidium bromide. To exclude non-specific PCR amplifications, the PCR products were then subjected to Southern blotting analysis with a radiolabelled 'nested' Ang1 primer that corresponded to a sequence of Ang1 cDNA between PCR primers. A positive signal for Ang1 was identified from RNA isolated from nine out of the 19 breast cancer cell lines.

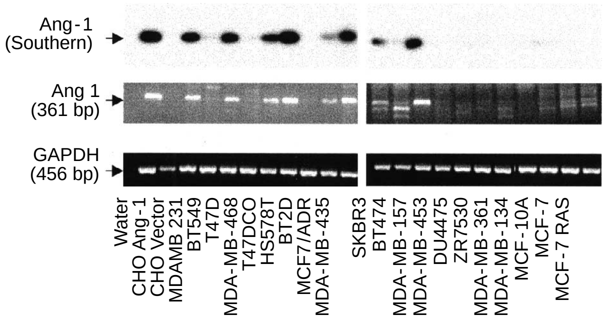

**Figure 1** RT-PCR analysis of breast cancer cell lines for Ang1 mRNA. Positive and negative control cells were CHO cells transfected with Ang1 (CHO Ang1) or an empty vector (CHO Vector). The lower two panels show RT-PCR products after agarose gel electrophoresis, visualized by ethidium bromide staining. The top row is a Southern hybridization of the Ang1 RT-PCR products with a $^{32}$P-labelled nested primer

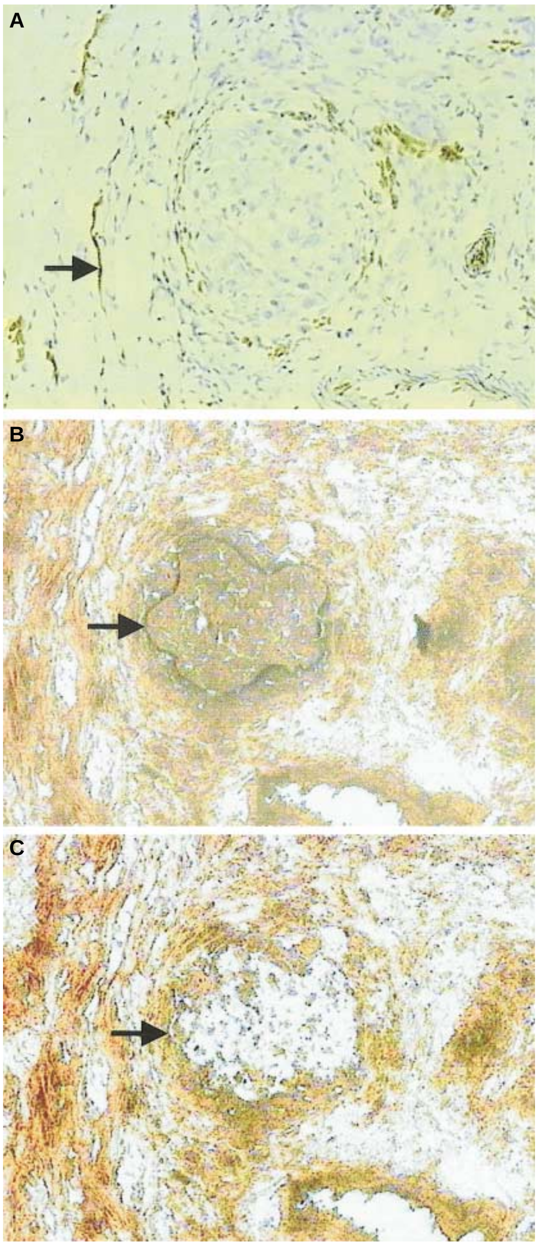

**Figure 2** Human breast cancer specimens before and after laser-capture microdissection. (**A**) Frozen section of ductal carcinoma was immuno-stained with a monoclonal antibody against Tie2: staining (arrow) indicates blood vessels that express Tie-2. (**B**) Serial section (eosin staining) showing the same ductule filled with breast cancer cells in which the cancer cells have been outlined by a laser field (arrow) prior to microdissection. (**C**) The same specimen as in **B** but subjected to microdissection; note the empty space left by the breast cancer cells now captured

### Low Ang1 expression in human breast cancer clinical specimens

We then determined whether breast cancer cells express Ang1 under clinical conditions. We utilized laser-capture microdissection and subsequent RT-PCR on the RNA isolated from microdissected specimens for this analysis. This technique permitted the isolation of a relatively homogeneous population of cancer cells adjacent to tumour vessels, which expressed a high level of the receptor Tie2 (Figure 2). It also involved amplification of the mRNA signal, as our previous efforts to determine Ang1 expression

 *© 2000 Cancer Research Campaign*

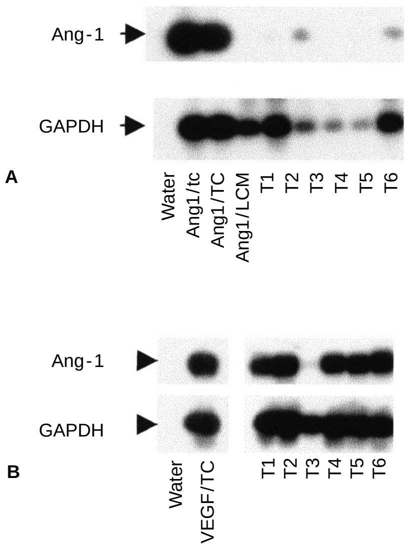

**Figure 3** Expression of Ang1 and VEGF in tumour specimens determined by using laser-capture microdissection. (**A**) Detection of Ang1 expression in six tumour specimens by RT-PCR and subsequent Southern analysis. Controls are RNA isolated from Ang1 transfected cells growing in culture (Ang1/TC) or microdissected from a section of a cell pellet (Ang1/LCM). (**B**) Detection of VEGF in the same cancer specimens by RT-PCR and Southern analysis with in situ hybridization experiment did not reveal any Ang1 signals in breast cancer specimens despite ample signals in control samples of Ang1-transfected cancer cells (data not shown). After reverse transcription of the mRNA from the microdissected cells, the cDNA samples were analysed for Ang1 by Southern blotting with a 'nested' oligonucleotide probe. VEGF mRNA, which is abundant in tumours, was analysed as a positive control. Figure 3 shows the results of an analysis of six tumour specimens. Control cells that expressed Ang1 display a clear signal for Ang1 which is approximately equal to that seen for GAPDH, an internal control, irrespective of whether the RNA was collected directly from cells growing in tissue culture or from frozen sections of cell pellets. In the majority of microdissection experiments no Ang1 signal was seen even after prolonged exposure, while the GAPDH signal was clearly demonstrated (Figure 3A). In contrast, similar analysis of VEGF expression in the same six tumour samples yielded a strong signal in five of six cases, confirming the abundance of this angiogenic factor in tumours and the suitability of microdissection as a method of analysis (Figure 3B).

Table 1 shows a summary of the results of Tie2 and Ang1 expression analysis of 11 normal and 23 malignant specimens. Samples were informative for Ang1 expression only if a GAPDH signal was visible after Southern hybridization. Although Tie2 is clearly expressed on microvessels of tumours and correlates closely to the expression of vWF (correlation coefficient 0.91), a detectable Ang1 signal was identified only in three of 21 tumour cases analysed and none of nine normal cases.

### Transfection of Ang1 cDNA into breast cancer cell lines results in the expression of high levels of biologically active Ang1 in conditioned media

In order to determine the effect of elevated Ang1 levels on tumour growth, we stably transfected Ang1 into two cell lines derived from the oestrogen-dependent, poorly angiogenic and weakly tumorigenic MCF-7 cells: the MPCX cells and the α18 cells. The MPCX cells, which were transfected with the β-galactosidase gene (lacZ) for convenient detection, maintains essentially the same growth characteristics to the MCF-7 cells, while the α18 cells, which were further transfected with FGF1, exhibited a greatly increased tumorigenicity and an extreme form of the dysfunctional vascular phenotype seen in xenograft tumours, with abundant vessels that are dilated (Zhang et al, 1997).

**Table 1** Summary of Ang1, Tie2, and vWF expression in clinical specimens. Clinical specimens (nine grade 3 tumours, nine grade 2 tumours, three grade 1 tumours and 11 normal breast sections) were stained with monoclonal antibodies to Tie2 and vWF. Positively staining vessels were counted manually using standard protocols (Weidner, 1995). Densities (±SEM) refer to number of vessels per 0.74 $mm^2$ microscopy field at $\times$ 200 magnification. Ang1 expression was analysed by laser-capture microdissection from cells adjacent to the immuno-stained vessels. Specimens were considered informative for Ang1 expression only if GAPDH was detectable

| Sample | Cases stained for Tie-2 and vWF | Number of Tie-2 expressing micro-vessels per sample | Number of vWF expressing micro-vessels per sample | Cases informative for Ang1 expression | Cases with Ang1 signal |
|---|---|---|---|---|---|
| Normal | 11 | 12 (±2.7) | 20 (±4.6) | 9 | 0 |
| Tumour | 23 | 35 (±3.7) | 44 (±3.9) | 21 | 3 |

Ang1 protein in the conditioned media of the transfected cells was detected by Western slot blot analysis using a polyclonal antibody against human Ang1 (Figure 4A). An estimation of the amount of Ang1 was made by comparison with signals from an Ang1 preparation of known concentration. The ability of the Ang1 protein to bind to Tie2 was determined by using a BIAcore analyser, which also allowed a quantitative determination of the concentration of Ang1 (Table 2). The Ang1 concentrations determined by these methods correlated closely. Furthermore, the biological activity of Ang1 in the conditioned media was determined by measuring the ability of the conditioned media to induce Tie2 tyrosine phosphorylation (Figure 4B). The extent of phosphorylation induced by conditioned media from the highest expressing clones was equivalent to that produced by 200 ng of recombinant Ang1, which gave rise to a maximum extent of phosphorylation of Tie2 on the cells. The α18-derived cell lines were further analysed for continued expression of FGF1 mRNA by Northern analysis (Figure 4C). All of the FGF1 transfected cell lines expressed high levels and equivalent amounts of FGF1 mRNA. In vitro mitogenesis assays were performed on all transfected and parental cell lines prior to animal inoculation, to ensure that the transfection procedure or the expression of Ang1 had not altered the in vitro growth characteristics (data not shown). As expected, Ang1 overexpression had no effect on the growth rates of all the selected clones in cultures since MCF-7 cells do not express the Tie2 receptor.

### Inhibition of MCF-7 xenograft tumour growth by Ang1 overexpression

Three Ang1 expressing clones, as well as a pooled population of empty vector transfected cells and the parental cells, were inoculated into the mammary fat pads of athymic nude mice. The growth rates of the xenograft tumours were monitored. The tumours were retrieved at the end of the experiment and the expression of Ang1 confirmed by Northern blotting analysis (data not shown). Although

*© 2000 Cancer Research Campaign* 

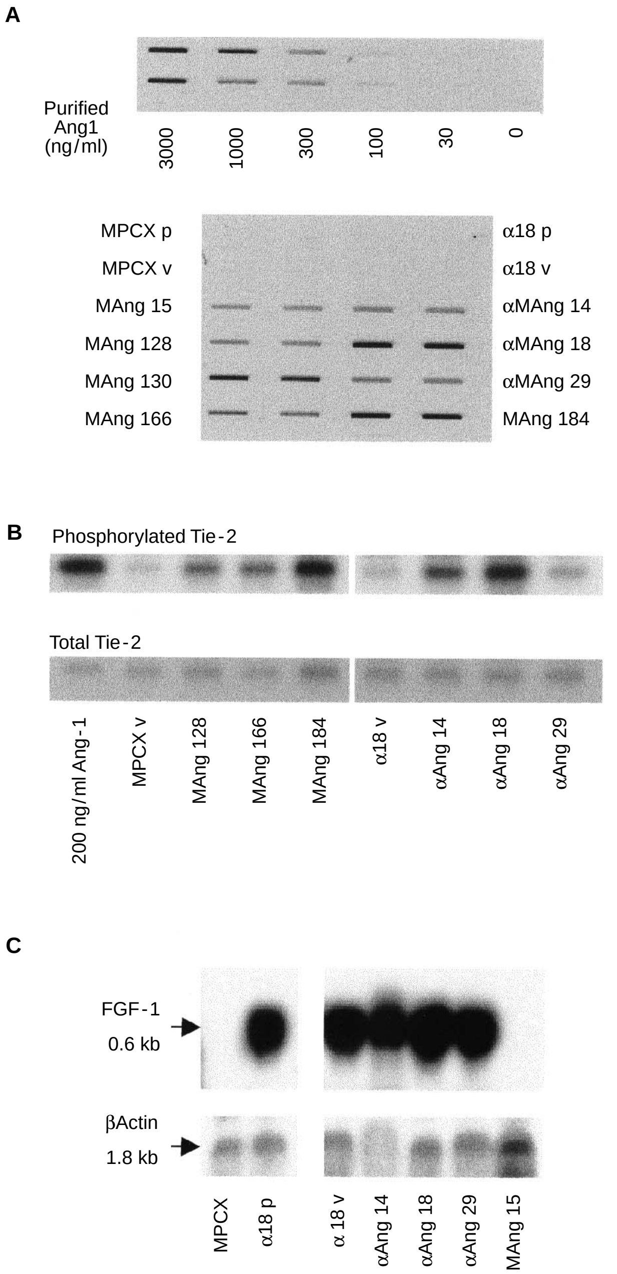

**Figure 4** Production of Ang1 by Ang1 transfected MPCX and α18 cells. (**A**) Western slot blot analysis of Ang1 in the conditioned media. Duplicate aliquots of COS cell conditioned media of known Ang1 concentration were used as standards (upper panel). Five clones of the Ang1 transfected MPCX cells and three clones of the Ang1 transfected α18 cells were shown in duplicate (lower panel), in comparison with the parental (P) and empty vector transfected (V) cells. (**B**) Ang1 activity in the conditioned media was determined for the ability to induce Tie2 tyrosine phosphorylation. NIH 3T3 cells overexpressing myc-tagged Tie2 were treated with the conditioned media, then subjected to immuno-precipitation with an anti-myc antibody, and Western blotting analysis with an anti-phosphotyrosine antibody (upper panel). The cells were also treated with 200 ng ml$^{-1}$ of Ang1 to provide a positive control. A Western analysis with an antibody against the myc-tag as a loading control (lower panel). (**C**) Northern blotting analysis of FGF1 in RNA collected from Ang1 transfected α18 cells. The internal control was β-actin FGF1

Ang1 overexpressing MPCX cells were able to grow xenograft tumours, the growth rates of the transfected cells were decreased as compared with the parental or vector control. A dramatic inhibitory effect ($P$ = 0.003) was observed with the clone MAng 184 that expressed the most Ang1 (Figure 5A). The extent of rate-decrease correlated reasonably well with the amount of Ang1 produced by the transfected cells (Figure 5B and Table 2).

**Table 2** Ang1 concentrations in the conditioned media of either vector transfected or Ang1 transfected cells (BIAcore analysis)

| Cell line | Ang1 (ng ml$^{-1}$) |
|---|---|
| MPCX Vector | 0 |
| MAng128 | 240 |
| MAng166 | 160 |
| MAng184 | 820 |
| α18 Vector | 0 |
| αAng14 | 172 |
| αAng18 | 612 |
| αAng29 | 156 |

Similar experiments were carried out with the FGF1 and Ang1 co-transfected α18 cell lines. The growth of the xenograft tumours of the Ang1 overexpressing cells was again found to be much slower than that of the parental cells and the vector mock transfected cells (Figure 6A). A statistically significant inhibition of tumour growth was observed with clones αAng 18 and αAng 29 ($P$ = 0.03). The dimensions of the tumours produced by clone αAng 14 were not statistically different from the parental or vector controls. However, for clone αAng 14 the recorded tumour volume did not represent the actual volume of tumour cells. This is because the majority of the volume of the xenografts formed by this clone at the end of the assay was caused by blood in a haemangectasic sac. In contrast, while the parental and vector cells produced a haemangectasic sac initially, this was replaced by a solid mass of tumour cells as the tumour growth progressed (Zhang et al, 1997). We demonstrated this at the end of the experiment by staining all of the xenografts with a β-galactosidase substrate. This allowed for easy identification of the tumour as blue cells (Figure 6B). A solid mass of cancer cells had replaced the haemangectasic sac in the tumours produced by the parental α18 cells (Figure 6B, right). In contrast, the haemangectasic sac responsible for the large dimensions of the αAng 14 tumours was still not replaced by a tumour mass at the end of the assay but consisted principally of blood (Figure 6B, left). On transection, it was demonstrated that the cancer cells occupied a relatively small proportion of the tumour volume (Figure 6B, centre) implying that the rate of tumour cell growth was very much smaller than that recorded by measurement of xenograft dimensions.

### DISCUSSION

We tested the hypothesis whether Ang1 would serve a pro-angiogenic role in the context of tumours, similar to its role suggested for physiological neovascularization. We found that Ang1 expression is lacking from areas adjacent to tumour blood vessels in human breast cancer despite abundant expression of Tie2 in these vessels. In addition, overexpression of Ang1 did not enhance xenograft growth and the majority of Ang1 transfected clones demonstrated decreased growth rates. Our data demonstrate that Ang1 may not function as a promoter of angiogenesis in breast tumours. Moreover, the data suggests that it may act in an inhibitory capacity in this model.

 *© 2000 Cancer Research Campaign*

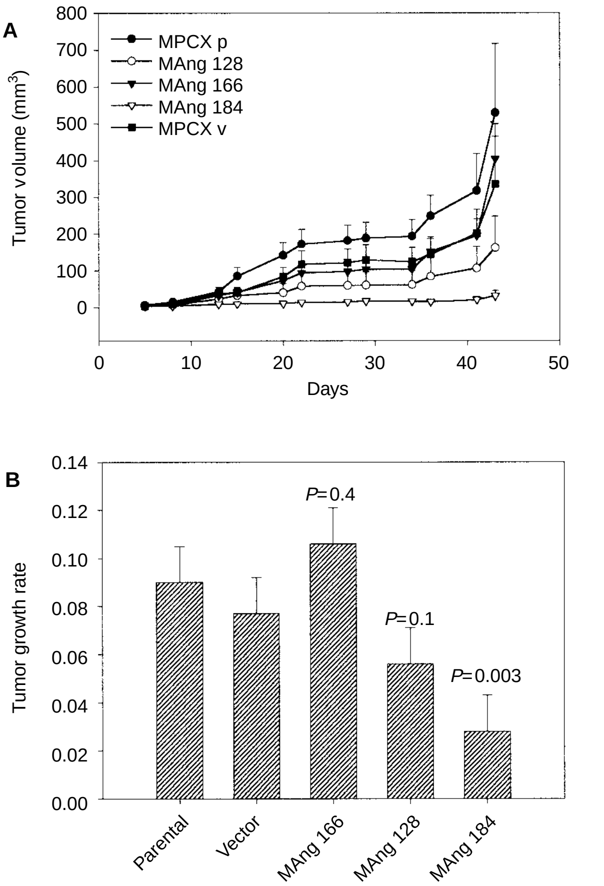

**Figure 5** Inhibition of MCF-7 human breast cancer xenograft tumour growth by Ang1 overexpression. (**A**) Plots of tumour volumes of the xenograft tumours formed by the MPCX parental cell line (closed circles), empty vector transfected cells (closed squares), and three clones of Ang1 expressing transfections: MAng 128 (open circles), MAng 166 (closed triangles), and MAng 184 (open triangles). There were five animals per group. The experiment was repeated and the results were reproducible. (**B**) The tumour sizes as a function of time were fitted with an exponential tumour growth model (see Methods) to determine the rate constants of the xenograft tumour growth. Growth rates for the Ang1-overexpressing MPCX clones were compared to that of the vector mock transfected cells (ANOVA)

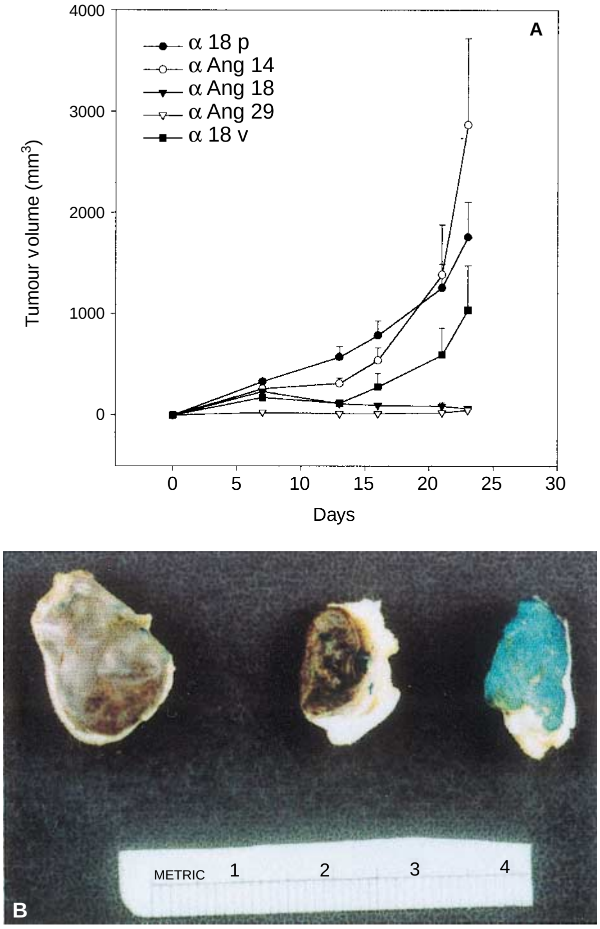

**Figure 6** Inhibition of FGF1 transfected MCF-7 breast cancer xenograft tumour growth by Ang1 overexpression. (**A**) Plots of tumour volumes of the xenograft tumours formed by the α18 parental cell line (closed circles), empty vector transfected cells (closed squares), and three clones of Ang1 expressing transfectants: αAng14 (open circles), αAng18 (closed triangles), and αAng29 (open triangles). The overall statistical significance at 5% level was $P$ = 0.03 (ANOVA) (n=5) for αAng18 and αAng29. The experiment was repeated and the results were reproducible. (**B**) Photographs of the xenograft tumours: a tumour formed by αAng14 giving the typical appearance of a blood filled sac (left), cross-section of a tumour in the same group showing that the interior of the sac is empty once opened, and that there are only a few cancer cells which were stained blue (centre), and the tumour formed by the parental α18 cells consists of blue cancer cells

These findings initially appear to be in contrast to transgenic studies in which Ang1 was overexpressed locally in the skin of developing mice. This overexpression of Ang1 induced remarkable increases in vascularity in the skin of these transgenic animals (Thurston, 1999). These seemingly contrary findings for Ang1 overexpression in transgenic animals and in our tumour models suggest that the process of tumour vascularization may be dissimilar to that seen in physiological settings. Physiological angiogenesis occurs in the context of a number of angiogenic factors and the expression of these factors is precisely coordinated both temporally and spatially (Dumont et al, 1995). Tumours elaborate a variety of angiogenic factors (Relf et al, 1997) and as a consequence of the abundance of angiogenic influences in the tumour micro-environment, the microvessel density in a tumour may be very high, although many vessels are dysfunctional. This dysfunction is manifested by areas of tumour necrosis and tumour hypoxia seen near to areas of increased microvessel density. The failure to identify Ang1 expression in the breast cancer epithelial cells suggests that Ang1, which is hypothesized to promote the ordered expansion of the vascular tree physiologically, may not be a pertinent angiogenic factor to the highly disordered tumour vasculature.

The data from overexpression of Ang1 in a xenograft model suggest that Ang1 may inhibit tumour growth, presumably via an effect on tumour angiogenesis, as Ang1 had no effect on the in vitro growth of the tumour cells. This is in keeping with the hypothesized roles of Ang1 and its functional antagonist Ang2 (Maisonpierre et al, 1997) on vessel stability and receptivity to other angiogenic influences. Ang1 stabilizes the association between the endothelial cell and pericyte (Suri et al, 1996; 1998; Thurston et al, 1999). Ang2, by antagonizing this effect, can result in vessel disassembly and subsequent vessel regression, but may, in the presence of other suitable angiogenic factors, facilitate new vessel sprouting (Maisonpierre et al, 1997). It has recently been observed that Ang2 is focally up-regulated in the immediate vicinity of tumour vessels (Stratmann et al, 1998; Zagzag et al, 1999). Therefore in tumours, where a variety of other angiogenic

*© 2000 Cancer Research Campaign* 

factors exist, this stabilizing effect of Ang1 might in fact inhibit the intense continuous new vessel sprouting that is typical of tumour vascularization. That Ang1 is able to inhibit tumour growth even in the presence of FGF1 supports the view that Ang1 retards tumour growth by vascular stabilization. Additionally, our findings that Ang1 is expressed in many breast cancer cell lines in vitro, but in very few clinical specimens, suggest that expression of Ang1 may be down-regulated in tumours because of its negative selective effect on the developing tumour.

To identify the mechanism by which Ang1 may lead to retardation of tumour growth requires a variety of further experimental approaches. These may include a detailed histological characterization of vessel branching and endothelial pericyte relations in the Ang1 overexpressing tumours and a direct in situ assessment of cellular proliferation rates within the xenografts expressing Ang1. It will be informative to assess the expression patterns of other ligands to Tie2 in clinical specimens, in particular Ang2. Such studies will further elucidate the role played by this complex vascular signalling pathway in the process of tumour vascularization.

### ACKNOWLEDGEMENTS

The authors thank Ms Mariella Tefft for assistance in statistical analysis. Research is supported in part by a grant DAMD17-97-1-7133 from the United States Department of Defence to LYL.

### REFERENCES

Asahara T, Chen D, Takahashi T, Fujikawa K, Kearney M, Magner M, Yancopoulos GD and Isner JM (1998) Tie2 receptor ligands, angiopoietin-1 and angiopoietin-2, modulate VEGF-induced postnatal neovascularization. *Circ Res* **83**: 233–240

Carmeliet P, Ferreira V, Breier G, Pollefeyt S, Kieckens L, Gertsenstein M, Fahrig M, Vandenhoeck A, Harpal K, Eberhardt C, Declercq C, Pawling J, Moons L, Collen D, Risau W and Nagy A (1996) Abnormal blood vessel development and lethality in embryos lacking a single VEGF allele. *Nature* **380**: 435–439

Davis S, Aldrich TH, Jones PF, Acheson A, Compton DL, Jain V, Ryan TE, Bruno J, Radziejewski C, Maisonpierre PC and Yancopoulos GD (1996) Isolation of angiopoietin-1, a ligand for the TIE2 receptor, by secretion-trap expression cloning. *Cell* **87**: 1161–1169

de Vries C, Escobedo JA, Ueno H, Houck K, Ferrara N and Williams LT (1992) The fms-like tyrosine kinase, a receptor for vascular endothelial growth factor. *Science* **255**: 989–991

Dumont DJ, Gradwohl GJ, Fong GH, Auerbach R and Breitman ML (1993) The endothelial-specific receptor tyrosine kinase, tek, is a member of a new subfamily of receptors. *Oncogene* **8**: 1293–1301

Dumont DJ, Fong GH, Puri MC, Gradwohl G, Alitalo K and Breitman ML (1995) Vascularization of the mouse embryo: a study of flk-1, tek, tie, and vascular endothelial growth factor expression during development. *Dev Dyn* **203**: 80–92

Emmert-Buck MR, Bonner RF, Smith PD, Chuaqui RF, Zhuang Z, Goldstein SR, Weiss RA and Liotta LA (1996) Laser capture microdissection. *Science* **274**: 998–1001

Ferrara N, Carver-Moore K, Chen H, Dowd M, Lu L, O'Shea KS, Powell-Braxton L, Hillan KJ and Moore MW (1996) Heterozygous embryonic lethality induced by targeted inactivation of the VEGF gene. *Nature* **380**: 439–442

Folkman J (1971) Tumor angiogenesis: therapeutic implications. *N Engl J Med* **285**: 1182–1186

Hanahan D and Folkman J (1996) Patterns and emerging mechanisms of the angiogenic switch during tumorigenesis. *Cell* **86**: 353–364

Hayes AJ, Huang WQ, Mallah J, Yang D, Lippman ME and Li LY (1999) Angiopoietin-1 and its receptor Tie-2 participate in the regulation of capillary-like tubule formation and survival of endothelial cells. *Microvasc Res* **58**: 224–237

Holash J, Maisonpierre PC, Compton D, Boland P, Alexander CR, Zagzag D, Yancopoulos GD and Wiegand SJ (1999) Vessel cooption, regression, and growth in tumors mediated by angiopoietins and VEGF. *Science* **284**: 1994–1998

Koblizek TI, Weiss C, Yancopoulos GD, Deutsch U and Risau W (1998) Angiopoietin-1 induces sprouting angiogenesis in vitro. *Curr Biol* **8**: 529–532

Kurebayashi J, McLeskey SW, Johnson MD, Lippman ME, Dickson RB and Kern FG (1993) Quantitative demonstration of spontaneous metastasis by MCF-7 human breast cancer cells cotransfected with fibroblast growth factor 4 and LacZ. *Cancer Res* **53**: 2178–2187

Lehtola L, Partanen J, Sistonen L, Korhonen J, Warri A, Harkonen P, Clarke R and Alitalo K (1992) Analysis of tyrosine kinase mRNAs including four FGF receptor mRNAs expressed in MCF-7 breast-cancer cells. *Int J Cancer* **50**: 598–603

Maisonpierre PC, Goldfarb M, Yancopoulos GD and Gao G (1993) Distinct rat genes with related profiles of expression define a TIE receptor tyrosine kinase family. *Oncogene* **8**: 1631–1637

Maisonpierre PC, Suri C, Jones PF, Bartunkova S, Wiegand SJ, Radziejewski C, Compton D, McClain J, Aldrich TH, Papadopoulos N, Daly TJ, Davis S, Sato TN and Yancopoulos GD (1997) Angiopoietin-2, a natural antagonist for Tie2 that disrupts in vivo angiogenesis. *Science* **277**: 55–60

McLeskey SW, Kurebayashi J, Honig SF, Zwiebel J, Lippman ME, Dickson RB and Kern FG (1993) Fibroblast growth factor 4 transfection of MCF-7 cells produces cell lines that are tumorigenic and metastatic in ovariectomized or tamoxifen-treated athymic nude mice. *Cancer Res* **53**: 2168–2177

McLeskey SW, Tobias CA, Vezza PR, Filie AC, Kern FG and Hanfelt J (1998) Tumor growth of FGF or VEGF transfected MCF-7 breast carcinoma cells correlates with density of specific microvessels independent of the transfected angiogenic factor. *Am J Pathol* **153**: 1993–2006

Papapetropoulos A, Garcia-Cardena G, Dengler TJ, Maisonpierre PC, Yancopoulos GD and Sessa WC (1999) Direct actions of angiopoietin-1 on human endothelium: evidence for network stabilization, cell survival, and interaction with other angiogenic growth factors. *Lab Invest* **79**: 213–223

Relf M, LeJeune S, Scott PA, Fox S, Smith K, Leek R, Moghaddam A, Whitehouse R, Bicknell R and Harris AL (1997) Expression of the angiogenic factors vascular endothelial cell growth factor, acidic and basic fibroblast growth factor, tumor growth factor beta-1, platelet-derived endothelial cell growth factor, placenta growth factor, and pleiotrophin in human primary breast cancer and its relation to angiogenesis. *Cancer Res* **57**: 963–969

Sato TN, Tozawa Y, Deutsch U, Wolburg-Buchholz K, Fujiwara Y, Gendron-Maguire M, Gridley T, Wolburg H, Risau W and Qin Y (1995) Distinct roles of the receptor tyrosine kinases Tie-1 and Tie-2 in blood vessel formation. *Nature* **376**: 70–74

Shalaby F, Rossant J, Yamaguchi TP, Gertsenstein M, Wu XF, Breitman ML and Schuh AC (1995) Failure of blood-island formation and vasculogenesis in Flk-1-deficient mice. *Nature* **376**: 62–66

Stratmann A, Risau W and Plate KH (1998) Cell type-specific expression of angiopoietin-1 and angiopoietin-2 suggests a role in glioblastoma angiogenesis. *Am J Pathol* **153**: 1459–1466

Suri C, Jones PF, Patan S, Bartunkova S, Maisonpierre PC, Davis S, Sato TN and Yancopoulos GD (1996) Requisite role of angiopoietin-1, a ligand for the TIE2 receptor, during embryonic angiogenesis. *Cell* **87**: 1171–1180

Suri C, McClain J, Thurston G, McDonald DM, Zhou H, Oldmixon EH, Sato TN and Yancopoulos GD (1998) Increased vascularization in mice overexpressing angiopoietin-1. *Science* **282**: 468–471

Takebe Y, Seiki M, Fujisawa J, Hoy P, Yokota K, Arai K, Yoshida M and Arai N (1988) SR alpha promoter: an efficient and versatile mammalian cDNA expression system composed of the simian virus 40 early promoter and the R-U5 segment of human T-cell leukemia virus type 1 long terminal repeat. *Mol Cell Biol* **8**: 466–472

Terman BI, Dougher-Vermazen M, Carrion ME, Dimitrov D, Armellino DC, Gospodarowicz D and Bohlen P (1992) Identification of the KDR tyrosine kinase as a receptor for vascular endothelial cell growth factor. *Biochem Biophys Res Commun* **187**: 1579–1586

Thurston G, Suri C, Smith K, McClain J, Sato TN, Yancopoulos GD and McDonald DM (1999) Leakage-resistant blood vessels in mice transgenically overexpressing angiopoietin-1. *Science* **286**: 2511–2514

Weidner N (1995) Current pathologic methods for measuring intratumoral microvessel density within breast carcinoma and other solid tumors. *Breast Cancer Res Treat*, **36**: 169–180

Zagzag D, Hooper A, Friedlander DR, Chan W, Holash J, Wiegand SJ, Yancopoulos GD and Grumet M (1999) In situ expression of angiopoietins in astrocytomas identifies angiopoietin-2 as an early marker of tumor angiogenesis *Exp Neurol* **159**: 391–400

Zhang L, Kharbanda S, Chen D, Bullocks J, Miller DL, Ding IY, Hanfelt J, McLeskey SW and Kern FG (1997) MCF-7 breast carcinoma cells overexpressing FGF-1 form vascularized, metastatic tumors in ovariectomized or tamoxifen-treated nude mice. *Oncogene* **15**: 2093–2108

*© 2000 Cancer Research Campaign*
